# Supplementary material for: Identification of photoperiod-induced specific miRNAs in the adrenal glands of Sunite sheep (Ovis aries)
Source: Front Vet Sci. 2022 Jul 22;9:888207. doi: 10.3389/fvets.2022.888207 (PMC9354845; doi:10.3389/fvets.2022.888207)
Supplement: Supplementary file 1 [file Data_Sheet_1.ZIP › Supplementary file/miRDeep2 file.zip!/miRDeep2 file/Know_Structure/SP42A2/oar-mir-379.pdf]

miRBase precursor : oar-mir-379
Total read count : 54798
oar-miR-379-5p read count : 51946
oar-miR-379-3p read count : 2843
remaining reads : 9

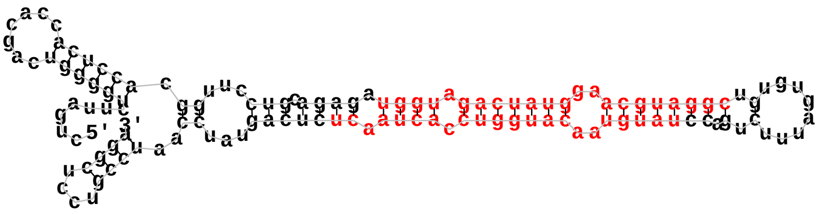

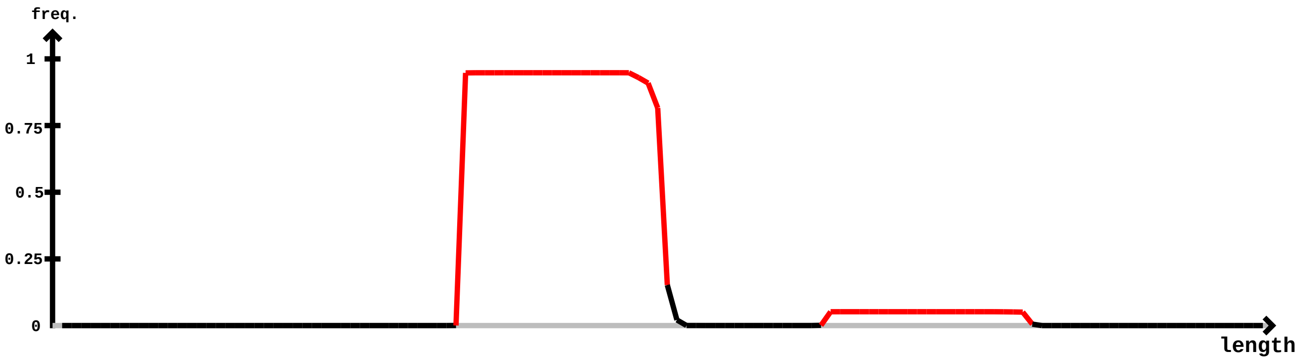

oar-miR-379-3p

oar-miR-379-5p

```
5'- cugauucuggggucagcaccacuccacgguuccugcagagaugguagacuauggaacguaggcugugugauuucugaccuauguaacaugguccacuaacucucaguauccaauccguccucgga -3'   exp
    .......((((((.......)))))).((...(((.((((.((((.(((((((..((((((((.(........).).)))))))..))))))).)))).)))))))...))..((((....))))   reads   mm
    .....................cuccacgguuccugcagaga....................................................................................   1       0
    ......................uccacgguuccugcagaga....................................................................................   1       0
    ........................................augguagacuauggaacgu..................................................................   1       0
    ........................................augguagacuauggaacguag................................................................   4       0
    ........................................augguagacuauggaacguagg...............................................................   8       0
    .........................................ugguagacuauggaacg...................................................................   1       0
    .........................................ugguagacuauggaacgu..................................................................   988     0
    .........................................ugguagacuauggaacgua.................................................................   1132    0
    .........................................ugguagacuauggaacguag................................................................   5106    0
    .........................................ugguagacuauggaacguagg...............................................................   36353   0
    .........................................ugguagacuauggaacguaggc..............................................................   7173    0
    .........................................ugguagacuauggaacguaggcu.............................................................   1146    0
    .........................................ugguagacuauggaacguaggcug............................................................   1       0
    .........................................ugguagacuauggaacguaggcugu...........................................................   2       0
    ..........................................gguagacuauggaacgua.................................................................   1       0
    ..........................................gguagacuauggaacguag................................................................   1       0
    ..........................................gguagacuauggaacguagg...............................................................   22      0
    ..........................................gguagacuauggaacguaggc..............................................................   4       0
    ..........................................gguagacuauggaacguaggcu.............................................................   1       0
    ...........................................guagacuauggaacguagg...............................................................   1       0
    .............................................agacuauggaacguagg...............................................................   1       0
    ..............................................................cugugugauuucugacc..............................................   6       0
    ..............................................................cugugugauuucugacU..............................................   1       1
    ..............................................................................cuauguaacaugguccacu............................   2       0
    ..............................................................................cuauguaacaugguccacua...........................   2       0
    ..............................................................................cuauguaacaugguccacuaa..........................   1       0
    ..............................................................................cuauguaacaugguccacuaac.........................   34      0
    ..............................................................................cuauguaacaugguccacuaacu........................   3       0
    ...............................................................................uauguaacaugguccacA............................   3       1
    ...............................................................................uauguaacaugguccacu............................   10      0
    ...............................................................................uauguaacaugguccacC............................   2       1
    ...............................................................................uauguaacauggucc Gcu............................   1       1
    ...............................................................................uauguaacaugguccacua...........................   25      0
    ...............................................................................uauguaacaugguccacuaG..........................   2       1
```

```
                                                                               oar-miR-379-3p
                                         oar-miR-379-5p
cugauucuggggucagcaccacuccacgguuccugcagagaugguagacuauggaacguaggcugugugauuucugaccuauguaacaugguccacuaacucucaguauccaauccguccucgga

...............................................................................uauguaacaugguccacuaa..........................    35      0
...............................................................................uauguaacaugguccacuaac.........................    2426    0
...............................................................................uauguaacaugguccacuaacu........................    293     0
................................................................................auguaacaugguccacuaac.........................    3       0
................................................................................auguaacaugguccacuaacu........................    1       0
```
